# A child with genetic FN1 mutation in the absence of classic glomerulopathy with fibronectin deposits(GFND) findings on biopsy

**DOI:** 10.1186/s12882-022-02872-x

**Published:** 2022-07-14

**Authors:** Xiao-qing Yang, Tong Shen

**Affiliations:** Pediatrics Department, Women and Children’s Hospital, School of Medicine, Xiamen University. Xiamen Maternal and Child Health Care Hospital, 361003 Xiamen, Fujian China

**Keywords:** Glomerulopathy with fibronectin deposits, Children, Hematuria, FN1, Alport syndrome

## Abstract

**Background:**

Glomerulopathy with fibronectin deposits (GFND) is a rare autosomal dominant genetic disorder, and proteinuria and hematuria are the most common clinical manifestations. The pathogenesis of this disease is primarily related to mutation of the fibronectin 1 gene. Unfortunately, without specific treatment, the prognosis remains poor. Here we present a case report that investigates the clinical characteristics, renal pathology, and gene testing of childhood GFND.

**Case presentation:**

A two-year-old child was brought to our hospital for “persistent hematuria for 1 year and 10 months.” The disease onset was at the age of 4 months, with persistent microscopic hematuria accompanied by intermittent gross hematuria, occasionally with proteinuria, and without hypertension or renal failure. The chief complaint was intermittent gross hematuria, without massive proteinuria, hypertension, or renal failure. Family history: The child’s mother had microscopic hematuria, his maternal aunt had nephrotic syndrome due to focal segmental glomerulosclerosis, and his maternal grandmother had end-stage renal disease. No significant pathological changes were found in the renal pathological biopsy of the child under a light microscope. Under the electron microscope, the basement membrane was found to be of uneven thickness, ranging from 150 to 400 nm. The stratum compactum of the basement membrane was thickened, with a small part showing tear-like and cobweb-like morphology. No electron-dense deposits were found. The renal tubular epithelial cells were vacuolated, and there were no unique pathological changes in the renal interstitium. Immunofluorescence showed that IgG, IgM, IgA, C3, and C1q were all negative. Alport syndrome was preliminarily considered. However, exome sequencing revealed a mutated site in the fibronectin 1 gene. The child’s mother was the carrier of the pathogenic gene and the final diagnosis was GFND.

**Conclusions:**

Fibronectin deposition is a typical pathological change in GFND, and the disease progresses slowly to end-stage renal disease. There is no specific treatment so far, and the prognosis is poor. The early onset of childhood patients may not show typical renal pathological changes, but only changes in the thickness of basement membrane, etc. Genome sequencing technology may helpful for the early diagnosis of GFND.

## Background

Glomerulopathy with fibronectin deposits (GFND) is an autosomal dominant glomerulopathy with age-related penetrance, characterised by fibronectin (FN) deposits within the glomeruli, the clinical manifestations of which are mainly proteinuria, microscopic hematuria, and hypertension [1, 2]. The predominant histological appearance of this disease is lobular, with mesangial PAS + deposits showing strong positive staining for fibronectin. Aetiology is unknown although several mutations in FN1 have been found in affected individuals [3, 4]. This paper reports a pediatric case with genetic FN1 mutation that urinary abnormalities occurred by the age of 2 years, the family pedigree was strongly consistent with autosomal dominant disease and kidney biopsy, performed early in life, did not show the typical changes, such as deposits.

## Case presentation

A boy, aged 2 years and 2 months, was admitted to our hospital on August 31, 2016, due to “microscopic hematuria found 1 year and 10 months ago, and intermittent gross hematuria for 2 months.” Microscopic hematuria was found during a health examination at the age of 4 months. Examinations showed: Urine protein 2+, occult blood 2+, urine microalbumin ≥ 100 mg/L. Urinary infection was initially diagnosed and Mezlocillin and sulbactam were administered for 5 days as an anti-infective therapy, and the hematuria improved upon reexamination. At the age of 6 months, the child underwent examinations, in which urine routine test showed 12–14 red blood cells/HP. Two months ago, the child intermittently discharged dark tea-colored urine. Urine routine test showed occult blood 3+. The child was referred to our hospital for further diagnosis and treatment. Physical examination showed no abnormal changes in the vision and hearing systems were observed in this child.

Family history was inquired upon admission: The child’s maternal grandmother had kidney failure by the age of 50 years, his maternal aunt had nephrotic syndrome with focal segmental glomerulosclerosis revealed by renal pathological biopsy at the age of 20, and his mother had a history of hematuria and nephrolithiasis for 5 years.

Laboratory examinations upon readmission included: Urine routine test: Urine occult blood 3+, red blood cells 1428.5/ul, protein (-); Urine red blood cell phase: Red blood cells 1,400,000/mL, percentage of malformed red blood cells 90.0–95%; blood albumin 36.7 g/L, total cholesterol 5.47 mmol/L, triglyceride 0.69 µmol/L, urea nitrogen 4.3 mmol/L, creatinine 27.6 µmol/L; 24-hour proteinuria 0.047 g/24 h, urine microalbumin 1.78 mg/dl. Coagulation function,immunoglobulin, complement, erythrocyte sedimentation rate and stool routine were normal; the 5 markers for hepatitis B, mycoplasma pneumoniae, EBV, and tuberculosis antibody were all negative. Color Doppler ultrasonography showed that the size of both kidneys was normal.

On the 6th day after admission, renal puncture biopsy was performed under the guidance of B-mode ultrasonography. Pathological results of renal biopsy included light microscope findings: Routine HE, PAS, PASM, and Masson staining was performed on the renal puncture biopsy tissue. A total of 18 glomeruli were observed. The mesangial cells and matrix did not proliferate significantly. There was no significant deposition of fuchsinophilic protein in the mesangial, subepithelial, or subendothelial region. The capillary loops were open, the basement membrane was not significantly thickened. Granular degeneration of renal tubular epithelial cells was found without significant atrophy. No significant inflammatory cell infiltration or fibrosis was found in the renal interstitium. No significant pathological changes of the arteriole walls were found. Electron microscope findings: The capillary endothelial cells were significantly vacuolated. Red blood cells could be seen in individual lumens. No significant endothelial cell proliferation was found. The capillary loops were open. The parietal layer of Bowman’s capsule was not significantly thickened or delaminated. The parietal cells were vacuolated. No significant hyperplasia was found; Basement membrane: The basement membrane was of uneven thickness, with a range of about 150–400 nm. The stratum compactum of the basement membrane was thickened, with a small part showing tear-like and cobweb-like morphology. Visceral epithelial cells: The epithelial cells were swollen and vacuolated, and phased fusion of foot processes was observed; Mesangial region: The mesangial cells and matrix had proliferated without electron-dense deposits. Renal tubules and interstitium: The tubular epithelial cells were vacuolated, while the renal interstitium was unremarkable; Renal interstitium blood vessels: Erythrocyte aggregation was seen in the lumens of individual capillaries, while no pathological changes were found in the arterioles. Immunofluorescence: IgG, IgM, IgA, C3, and C1q were all negative.

**Fig. 1:**
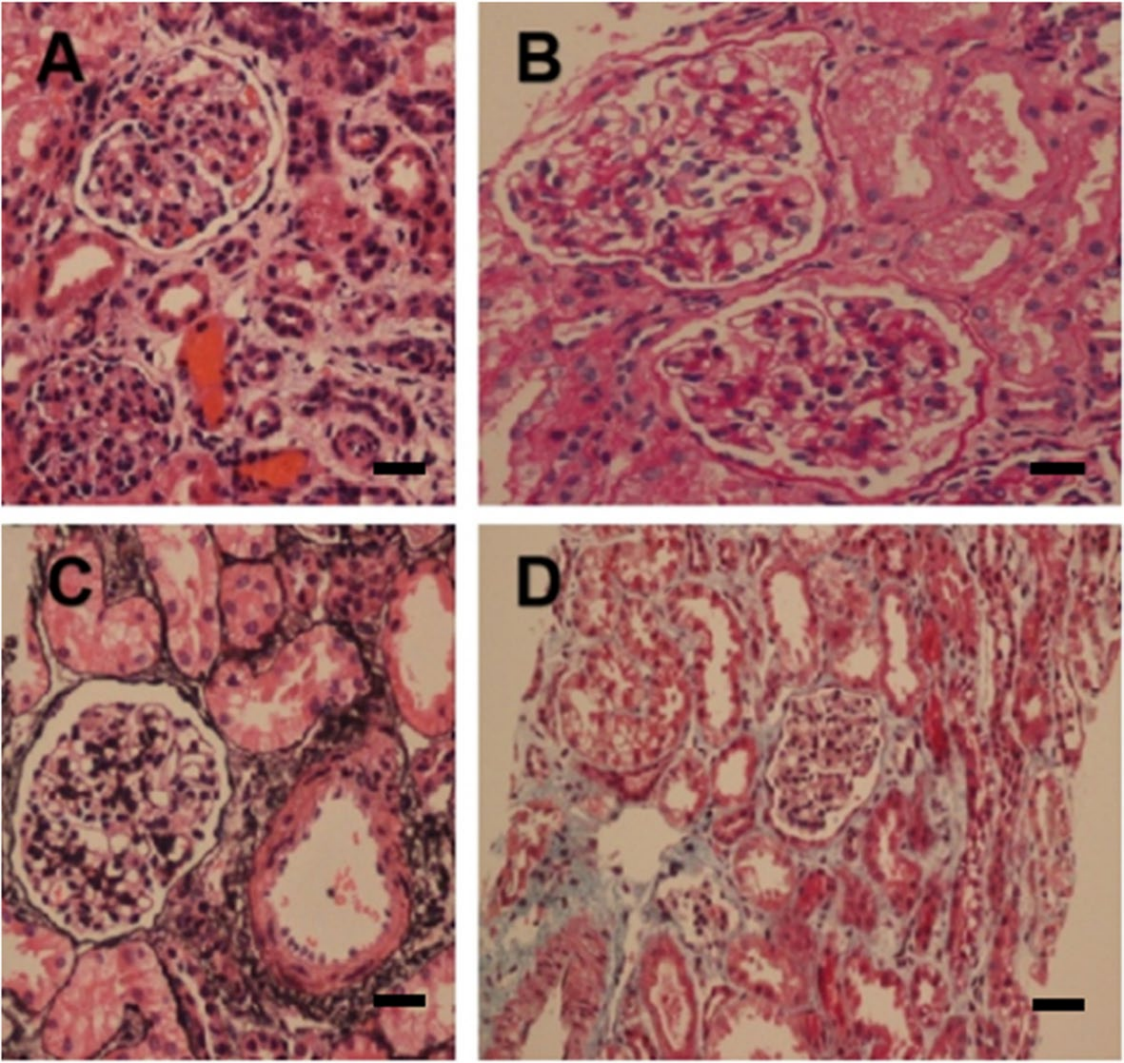
Pathological results of renal biopsy. **A** HE staining; **B** PAS staining; **C** PASM staining; **D** Masson staining. (Scale bars: A-D：10×400)

**Fig. 2:**
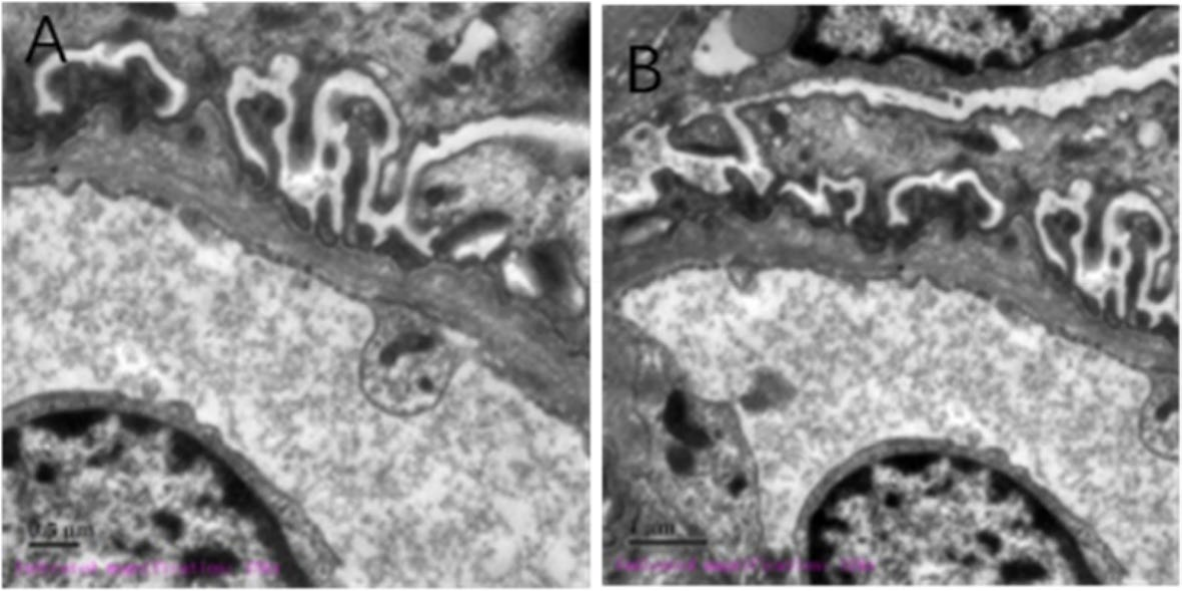
Electron microscope pathological results. (Scale bars: **A **0.5μm; **B **1μm)

**Fig. 3:**
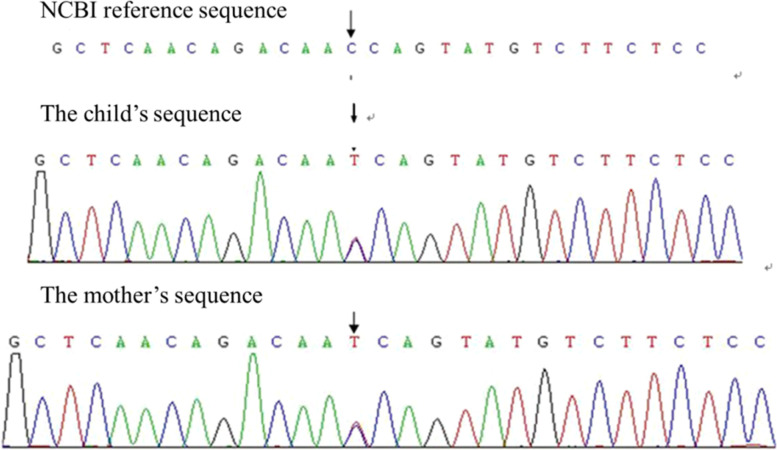
Sequencing results

**Fig. 4:**
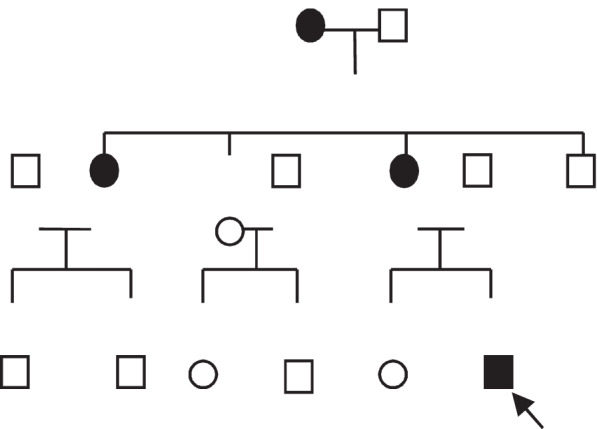
Genogram

Taking into consideration these findings, renal injury from Alport syndrome was considered. The pathology slides are shown in Fig. 1 A and B (light microscope HE and PAS staining, 10 × 400) and C and D (light microscope Periodic Schiff-Methenamine (PASM) and Masson staining, 10 × 400). Figure 2 is shown Under the electron microscope, the basement membrane was of uneven thickness, with a range of about 190–420 nm. Results of whole-exome sequencing: Whole-exome sequencing was performed on the child’s blood using the genome analysis method of high-throughput sequencing. The results showed a heterozygous mutation of the FN1 gene at chr2:216270963 with c.2984 C > T (exon19) and p.T995I. Further examination of the child’s mother showed the same heterozygous mutation of the FN1 gene. Given the clinical findings and the family history, the detected variant was considered compatible with GFND. Genetic testing for alports is normal. No mutation in COL4A5 gene was found. The sequencing results are shown in Fig. 3 and genogram is shown in Fig. 4.

## Discussion

Alport syndrome, also known as hereditary nephritis, is characterized by hematuria, proteinuria, and progressive renal failure. Some patients may suffer from extrarenal symptoms such as sensorineural hearing loss and vision loss [5]. The COL4A5 gene mutation leads to progressive and irregular thickening, thinning, or splitting of the glomerular basement membrane, which is the basis for the genetic and renal histopathological diagnosis of this disease. In this case, the child’s disease onset was at 4 months after birth, with persistent microscopic hematuria, intermittent proteinuria, gross hematuria, Upon inquiry, a family history of renal disease was discovered. The child’s maternal grandmother was diagnosed with uremia at the age of 50. His maternal aunt experienced proteinuria at the age of 20, with renal pathological diagnosis of focal segmental glomerulosclerosis. The child’s mother had a 5-year history of hematuria (see Fig. 4 for the Genogram). At the same time, renal tissue histopathology of the child under an electron microscope showed an uneven thickness of the glomerular basement membrane, and thickening of the stratum compactum with tear-like and cobweb-like morphology in areas, suggesting a high possibility of Alport syndrome. However, no abnormal changes in the vision and hearing systems were observed in this child. Unfortunately, the child did not undergo basal membrane IV collagen beta immunofluorescence test. In order to confirm the diagnosis, we performed whole-exome sequencing. The results revealed no mutation of the COL4An gene. Instead, site mutation of the fibronectin 1 (FN1) gene had occurred, i.e., heterozygous mutation at chr2:216270963 with c.2984 C > T (exon19) and p.T995I. Therefore, genetic finding appeared inconsistent with Alport syndrome and compatible with GFND.

First reported by Tuttle et al. in 1987, GFND is a rare hereditary glomerulopathy newly recognized in recent years [1]. The genetic disease is autosomal dominant, but the exact pathogenesis of the disease remains unclear. Proteinuria and hematuria are the primary clinical manifestations and symptoms can persist for decades. At present, however, there are still few reports on it in the literature from China and the rest of the world [6–8]. As a result, diagnosis and treatment remain challenging. Some patients present with hypertension and slow-progressing glomerular filtration rate decline, and even progress to renal failure. Among the reported cases, the youngest was aged 3 years and the eldest was 88 years [9, 10]. The child reported here was only 2 years and 2 months, which may be the youngest confirmed case reported so far, suggesting that the disease may occur in any age group.

The typical histopathological changes of GFND are glomerular lobular pattern, mesangial and GBM expansion, double contour and strongly positive fibronectin, and deposition of a large amount of fine fibrous materials can be observed under electron microscope. The child in this case had not shown the above-mentioned typical pathological changes yet. Considering that the child mainly manifested and proteinuria only occurred intermittently, the onset age of the child was young, the duration of the medical history was short, and typical pathological changes might not have occurred, it was suggested that long-term follow-up should be conducted for the child, and renal biopsy should be performed again as necessary to confirm the pathological diagnosis.

The key pathogenesis of GFND is the deposition of fibronectin (FN) in glomeruli. At present, it is believed that the impaired ability of glomeruli to process deposits due to the FN1 gene defect is an important cause of GFND. The FN1 gene is located on chromosome 2 and the main mutation point. The abnormality of FN1 gene in this child was manifested by the substitution of cytosine by thymine in the base pair at position 2,984 of exon 19 [c.2984 C > T(exon19)] in the gene coding sequence of chromosome 2 (position: chr2:216270963); this caused the missense mutation of the amino acid at position 995 of the transcribed mRNA from threonine to isoleucine (p.T995I). This mutation simultaneously affects the interaction of the heparin-binding domain among fibronectin cells on the one hand and while promoting the formation of fibronectin on the other hand. In 2016, Ohstubo et al. [3] conducted the largest pedigree analysis to date for patients with GFND, showing that 6 types of FN1 gene mutations were found in 12 GFND pedigrees, and more mutant genes have continuously been found. In addition to the gene reported in this case, pathogenic FN1 gene mutations found in China and other countries also include p.T973C, p.1T925A, p.T1925C, p.L1974A, p.P969L, and p.1P974D. In this case, the genetic diagnosis was made before the typical renal pathological changes appeared, suggesting the advantages of early-stage genetic diagnosis and providing conditions for early intervention.

Currently, there is no effective or specific treatment for GFND, and only symptomatic treatment is available, in which hormonal and immunosuppressive therapies have no significant efficacy, with poor prognosis. Literature shows that even after kidney transplantation, the disease can still relapse [11]. The child did not receive special treatment after discharge from the hospital. Follow-up was conducted for 3 years and 4 months, which showed that he still had persistent microscopic hematuria and intermittent proteinuria, while no abnormality in renal function was found. We suggested he return to the hospital for reexamination and another kidney biopsy, but his family refused.

The limitation of this study is that, on the one hand, fibronectin and collagen were not stained in the biopsies of the children and their aunt, which affects the diagnosis of this disease to a certain extent. On the other hand, the impact of the disease on the children cannot be assessed without functional analysis of the detected mutations.

## Conclusions

In summary, GFND is an autosomal dominant genetic disease with familial inheritance. This disease should be considered when patients present with clinical manifestations of proteinuria, hematuria, hypertension, and slow progression of renal hypofunction accompanied by a family history. Renal biopsy is of vital importance to the diagnosis. The key pathogenesis of GFND is the deposition of FN in glomeruli. Genome sequencing technology may be helpful for the early diagnosis of GFND.

## Data Availability

Reasonable requests for access to the datasets used and/or analyzed during the study can be made to the corresponding author.

## Abbreviations

GFND: Glomerulopathy with fibronectin deposits
FN1: Fibronectin 1
AD: Autosomal dominant
PASM: Periodic Schiff-Methenamine
